# Oral Hygiene a Necessity to Health

**Published:** 1906-08-15

**Authors:** Burton Lee Thorpe


### ORAL HYGIENE A NECESSITY TO HEALTH

BY BURTON LEE THORPE, M.D., D.D.S.

The medical profession and the layman seemingly do not realize the importance of the hygienic care of the oral cavity which is the incubator of many of the systemic diseases that the human system is heir to.

Frequent cases of disease of the intestinal tract ,such as dyspepsia and gastritis, may primarily be traced to infection caused by decayed teeth and defective hygiene of the mouth due to the accumulation of salivary calculus (tartar), the remains of diseased roots of teeth, which cause abscesses, hypertrophied (spongy) gums, etc., all of which contribute to systemic infection.

Some cases of appendicitis no doubt result from unhygenic conditions of the mouth. The writter has seen one appendix which contained an amalgam filling, several bird shot and several bits of calcic material apparently broken loose from around the teeth and swallowed.

It is a general accepted belief that *the mouth is the breeding place* of the *bacillus of influenza, and that oral sepsis is the predisposing cause of influenza, and that patients having hygienic mouths are practically immune from influenza.*

In diagnosis it is the duty of the medical practitioner to examine the condition of his patients' teeth and gums, and if carious teeth, inflamed and spongy gums, roots and their sequelæ, *i. e.*, abscessed conditions, etc., are in evidence they should be immediately referred to the dental practitioner for treatment. No greater service can be rendered the average man, woman or child than, at least, regular semi-annual visits to the dentist, who should remove the salivary deposits, thoroughly polish all surfaces of the teeth, treat and cure hypertrophied gums and repair all defects resulting from caries, with instruction of when and how to brush the teeth and gums and the prescribing of a proper tooth brush and antiseptic mouth-wash.

Few patients know that the teeth and gums should be brushed immediately upon rising in the morning and again just prior to retiring at night and after each meal where it is possible. The brushing and cleansing of all mucuos tissue *i. e.*, gums, tongue, buccal and labial cheek and lip muscles, and the palate tissue, is of equal value and importance. This may be accomplished by a *stiff bristled flat* surface tooth brush, with which the gums are massaged with a rotary motion, brushing the lower gums upward and the upper gums downward toward the neck of the teeth.

The commonly used tooth brush with a curved handle and irregular rows of bristles should be discarded, as *it does not* remove the deposits of bacteria and food debris lodged around the tooth and gum surfaces, as does the flat surface brush.

Another useful instrument indicated for patients who continually possess "the constipated" furred tongue, is the tongue scrapper—a narrow celluloid band curved—about the size of a silver half-dollar, attached to a celluloid handle. With this instrument drawn across the tongue daily the hardened fur-like mucus can be scraped from the tongue, thus greatly increasing the hygiene of the mouth. Floss silk or small rubber bands run between the surfaces of the teeth after eating also materially aids in lessening the chance of decay and oral sepsis.

The dentist is best prepared to prescribe a proper mouth wash after testing the saliva; however, one simple and beneficial to the majority of mouths, which hardens the gums and cleanses the teeth, is none other than a two per cent carbolic acid solution held in the mouth during the process of brushing.

In mouths where the saliva is acid, of course an alkaline mouth wash is needed, such as a solution of milk of magnesia applied nightly. As a preventive of decay and an agent to harden diseased gum tissue, a ten per cent solution of silver nitrate is unequalled. This, however, should be applied only by the dentist at intervals, as it temporarily stains the teeth and should be removed by him at a following visit with brushes and powders.

The main essentials are the mechanical and surgical removals of all deposits and diseased tissue, followed by astringent and antiseptic agents to heal the gum tissue, and the constant practise by the patient of daily mechanical friction with brush, powder and antiseptic washes. With these general infection may be avoided and systemic diseases prevented.—*Medical Brief, July* 1906.

